# Diagnosis of peri-prosthetic loosening of total hip and knee arthroplasty using ^18^F-Fluoride PET/CT

**DOI:** 10.18632/oncotarget.26762

**Published:** 2019-03-15

**Authors:** Sebastian Koob, Florian C. Gaertner, Tom Rainer Jansen, Jan Schmolders, Sascha Gravius, Holger Strunk, Dieter Christian Wirtz, Markus Essler

**Affiliations:** ^1^ Clinic for Orthopedics and Trauma Surgery, University of Bonn, Bonn, Germany; ^2^ Clinic for Nuclear Medicine, University of Bonn, Bonn, Germany; ^3^ Department of Radiology, University of Bonn, Bonn, Germany

**Keywords:** periprosthetic loosening, bone imaging, total hip arthroplasty, periprosthetic infection, ^18^F-Flouride PET/CT

## Abstract

Periprosthetic loosening, either aseptic or induced by periprosthetic joint infection remains a major long term complication and challenge in orthopedics and trauma surgery. Sensitivity of potential loosening of the material and other causes of a painful prosthesis is essential for choosing the respective treatment option and providing the needed resources. ^18^F-Fluoride is a radiopharmaceutical which shows a high affinity to bone and a rapid blood clearance.

The objective of this study was to assess ^18^F-Fluoride PET/CT´s sensitivity and specificity in diagnosing periprosthetic loosening in total hip and knee arthroplasty.

We included 26 patients with 24 hip and 13 knee prostheses in our retrospective study with radiological or clinical suspicion of peri-prosthetic loosening at least one year after implantation. Results of ^18^F-Fluoride PET/CT imaging were compared with surgical results or clinical follow-up if surgery was not performed. On the basis of our data we found a sensitivity of 95.00 %, a specificity of 87.04 % and an accuracy of 89.19 % for ^18^F-Fluoride PET/CT.

The results of our study show that ^18^F-Fluoride PET/CT is a useful and promising technique in diagnosing periprosthetic loosening of total hip and knee arthroplasties. Further investigation should focus on different uptake patterns of the isotope in periprosthetic joint infection and therefore distinguishing aseptic from septic loosening and enhancing the diagnostic value of this imaging method.

## INTRODUCTION

Total hip and knee arthroplasties in end-stage arthritis contribute an essential number to today´s orthopedic procedures. Periprosthetic loosening, either aseptic or induced by periprosthetic joint infection remains a major long term complication and challenge in orthopedics and trauma surgery. According to estimations between 0.4 and 4% of joint replacements will face periprosthetic infection and 2-18 % aseptic loosening [1]. When dealing with painful prostheses an early distinguishment between potential loosening of the material and other causes is essential for choosing the respective treatment option and providing the needed resources [2].

According to current understanding a chronic inflammatory process of the bone- prosthesis or cement-prosthesis interface causes implant loosening. Wear particles are being phagocytosed by macrophages which induce chronic inflammation and osteolysis with activation of osteoblastic and osteoclastic activity [3, 4]. Early before the gross implant motion sets in and radiographic changes can be recognized, so called micromotion already causes pain and makes diagnosis difficult. Today´s standard diagnostic procedures for ruling out prosthetic loosening most often include x-ray and bone scan imaging, as well as joint punctures and laboratory tests which feature wide ranges in specificity and sensitivity [1]. Especially plain radiographs often show signs of loosening only after a long delay of time. Traditional bone scan imaging comes along with a low spatial resolution.

^18^F-Fluoride PET/CT provides a promising alternative. ^18^F-Fluoride is a radiopharmaceutical which shows a high affinity to bone and a rapid blood clearance with high bone-to-background ratio in a shorter time than for standard ^99m^Tc-based tracers [5, 6]. In combination with positron emission tomography and its excellent spatial resolution ^18^F-Fluoride PET/CT may offer highly valuable images for detecting loose components of total hip and knee arthroplasties.

Therefore, the objective of this study was to assess ^18^F-Fluoride PET/CT´s sensitivity and specificity in diagnosing periprosthetic loosening in total hip and knee arthroplasty.

## RESULTS

The collective of 26 patients included 15 male and 11 female patients with a mean age of 67.5 years (50.1 - 92.2 years). We evaluated a total of 24 hip and 13 knee prostheses. Since the high resolution of the imaging allows for a separate evaluation of the femoral, tibial and acetabular part of an arthroplasty we were able to analyze 74 prosthetic components. The mean time between implantation and ^18^F-Fluoride PET/CT scan was 6.5 years (range 1.0 – 19.6 years).

The clinically silent additional 11 prostheses (22 components) were counted as true negative since the ^18^F-Fluoride PET/CT showed no signs of loosening.

For 18 patients and therefore 18 prostheses (36 components, 48.6%) the ^18^F-Fluoride PET/CT diagnosis was confirmed or disproved by intraoperative findings. In these cases the surgeon declared the respective components as loose or stable as stated in his operational report.

For 19 prostheses (38 components, 51.4%) without indication for revision surgery a clinical follow up lasting at least 6 months confirmed the ^18^F-Fluoride PET/CT diagnosis of a loosened or non loosened prosthesis.

One patient did not undergo revision surgery of two total knee arthroplasties due to age and the high risk profile of the operation although showing distinctive signs of prosthetic loosening on both sides. The clinical und radiological follow up for one year confirmed the diagnosis of loosened prostheses and its progression. These cases were therefore evaluated as true positive.

One patient with a positive ^18^F-Fluoride PET/CT finding for a loosening of the tibial component of the total knee arthroplasty has not been operated and showed no clinical symptoms in the follow up counting as false positive.

^18^F-Fluoride PET/CT correctly identified 18 out of 19 loosened total hip and knee arthroplasty components. In 6 out of 55 components without intraoperative or clinical signs of loosening we found a positive ^18^F-Fluoride PET/CT enhancement around the prosthesis counting as false positive. The others were correctly identified as not loosened in the scan.

On the basis of these data and considering every single prosthesis component (total 74, view Table 1) we found a sensitivity of 95.00 %, a specificity of 87.04 % and an accuracy of 89.19 %. These data include the clinically silent additional 11 prostheses (22 components).

**Table 1:** Included patients with total hip arthroplasty (THA) and total knee arthroplasty (TKA)

Patient	Prosthesis	Sex	Age (y)	Joint	Result of ^18^F-Fluoride PET/CT	Follow-up	Acetabular Component	Femoral Component	Tibial Component	Comment
1	1	M	69	**THA (right)**	Femoral Loosening	Surgery	TN	TP	-	Exchange of femoral component
	2			THA (left)	No Loosening	Clinical	TN	TN	-	No clinical symptoms
2	3	F	72	**THA (right)**	Acetabular loosening	Surgery	TP	TN	-	Exchange of Prosthesis
	4			THA (left)	No loosening	Clinical	TN	TN	-	No clinical symptoms
3	5	M	83	**THA (left)**	No loosening	Clinical	TN	TN	-	Spine-related symptoms
	6			THA (left)	No loosening	Clinical	TN	TN	-	No clinical symptoms
4	7	M	68	**TKA (right)**	Femoral loosening	Surgery	-	TP	TN	Exchange of femoral component
5	8	M	86	**THA (left)**	Femoral and acetabular loosening	Surgery	TP	FP	-	Exchange of acetabular component
6	9	M	73	**THA (right)**	Femoral and acetabular loosening	Surgery	TP	TP	-	Exchange of Prosthesis
7	10	F	65	**TKA (right)**	Tibial loosening	Clinical	-	TN	FP	Regression of symptoms after PT
8	11	F	54	**THA (left)**	Acetabular loosening	Surgery	TP	TN	-	Exchange of Acetabular Cup
9	12	F	55	**THA (left)**	No loosening	Surgery	TN	TN	-	Peri-articular ossifications
10	13	F	80	**THA (right)**	No loosening	Surgery	TN	TN	-	Tractus Gap
	14			TKA (left)	No loosening	Clinical	-	TN	TN	No clinical symptoms
11	15	F	75	**THA (left)**	No loosening	Surgery	TN	FN	-	Exchange of femoral component
	16			THA (right)	No loosening	Clinical	TN	TN	-	No clinical symptoms
12	17	M	64	**THA (right)**	Femoral and acetabular loosening	Surgery	FP	TP	-	Exchange of femoral component
	18			THA (left)	No loosening	Clinical	TN	TN	-	No clinical symptoms
13	19	F	92	**TKA (right)**	Femoral and tibial loosening	Clinical	-	TP	TP	Patient refused surgery
	20			TKA (left)	Femoral and tibial loosening	Clinical	-	TP	TP	Patient refused surgery
14	21	M	55	**THA (left)**	Femoral loosening	Surgery	TN	TP	-	Exchange of femoral component
15	22	F	67	**TKA (left)**	Femoral and tibial loosening	Surgery	-	TP	TP	Exchange of Prosthesis
16	23	F	54	**TKA (left)**	Femoral and tibial loosening	Surgery	-	FP	TP	Exchange of Prosthesis
17	24	M	50	**THA (right)**	No loosening	Clinical	TN	TN	-	Regression of symptoms after PT
	25			THA (left)	No loosening	Clinical	TN	TN	-	No clinical symptoms
18	26	M	76	**THA (left)**	No loosening	Clinical	TN	TN	-	Regression of symptoms after PT
19	27	M	63	**THA (left)**	Femoral and acetabular loosening	Clinical	TP	TP	-	Patient refused surgery
20	28	M	63	**THA (left)**	Femoral and acetabular loosening	Surgery	FP	TP	-	Exchange of femoral component
21	29	F	74	**TKA (right)**	Femoral loosening	Clinical	-	FP	TN	Regression of symptoms after PT
	30			TKA (left)	No loosening	Clinical	-	TN	TN	No clinical symptoms
	31			THA (left)	No Loosening	Clinical	TN	TN	-	No clinical symptoms
22	32	M	82	**TKA (right)**	No Loosening	Surgery	-	TN	TN	Patellar surface
23	33	F	53	**TKA (left)**	No loosening	Surgery	-	TN	TN	Patellar surface
24	34	M	71	**TKA (right)**	No Loosening	Surgery	-	TN	TN	Patellar surface
25	35	M	58	**TKA (left)**	Tibial loosening	Surgery	-	TN	FP	Patellar surface
26	36	M	66	**THA (left)**	No Loosening	Clinical	TN	TN	-	Soft tissue - related symptoms
	37			THA (right)	No Loosening	Clinical	TN	TN	-	No clinical symptoms

TN = True Negative, TP = True Positive, FN = False Negative, FP = False Positive, PT = Physiotherapy, bold = clinically suspicious prostheses.

When considering the respective endoprosthesis as one compound without distinguishing the femoral, tibial or acetabular component (n=37, view Table 1), sensitivity lowers to 92.86 % with a specificity of 86.96 % (accuracy = 89.19 %). Since indication for revision surgery is given if at least one component of the prosthesis is being detected as loose, this perspective rather reflects clinical reality.

When considering only the painful and clinically suspicious prostheses (26, view Table 1, marked bold), which led to the patient undergo diagnostics, sensitivity is 92.31 % and specificity lowers to 76.92 % (accuracy = 84.62 %).

## DISCUSSION

The aim of this study was to evaluate the role of ^18^F-Fluoride PET/CT in diagnosing periprosthetic loosening in total hip and knee arthroplasty.

M. Blau et al. were the first to use Fluorine-18 in bone imaging in 1962 [7], whereas Creutzig et al. presented a study of 31 hip prosthesis which he examined towards loosening and infection using planar ^18^F-Fluoride scanning in 1976 [8]. Since then little research has been performed regarding this radiopharmaceutical and prosthesis loosening. Nevertheless, other impairments of bone metabolic activity have been very well investigated using ^18^F-Fluoride PET, including renal osteodystrophy [9], osteoporosis [10], osseointegration of bone allografts [11], femoral head necrosis [12] and even bone malignancies [13].

In 2006 Sterner et al. showed in a study of 14 knee prostheses a sensitivity of 100% for diagnosing aseptic loosening with ^18^F-Fluoride PET/CT, whereas specificity was very low with 56% and the relatively high rate of false positive results could not be explained by the authors [14]. Still, they were the first to present results of ^18^F-Fluoride PET imaging in diagnosis of aseptic loosening. Compared to the above mentioned study our ^18^F-Fluoride PET/CT data show both a high sensitivity and superior specificity.

The so far most investigated radionuclide procedure used for imaging joint arthroplasties is bone scintigraphy with Technetium-99m (^99m^Tc) labeled diphosphonates, usually methylene diphosphonate (MDP). No matter what type of protocol or evaluation has been used, the accuracy of the method never exceeded 70%, whereas specificity has always been reported low, mostly due to the limitations in spatial resolution and therefore evaluation of distinctive uptake patterns of the isotope [15–18]. ^18^F-Fluoride PET/CT however shows an excellent resolution enabling a more accurate observation of the pattern resulting in a higher specificity of the diagnosis.

In 2011 Choe et al. presented a comparatively large prospective trial towards differentiation between septic and aseptic loosening by using ^18^F-Fluoride PET/CT including 49 patients with 65 total hip arthroplasties. To our knowledge this represents the first prospective investigation in this regard [19] and the highest number of participants. The method involved measurement of the degree of ^18^F-Fluoride uptake in periprosthetic tissue for septic and aseptic loosening. Our study did not include these types of measurements, but since differentiation between aseptic and septic loosening is key to an adapted treatment, further investigation in this matter is warranted.

Our and the above mentioned studies included patients whose prostheses have been implanted at least 1 year prior to ^18^F-Fluoride PET/CT imaging. According to Creutzig et al. unspecific ^18^F-Fluoride uptake especially around the acetabular component of total hip arthroplasty fades out about 9 months after implant surgery [8]. This is an argument in favor of using ^18^F-Fluoride for this indication, considering that fluorodeoxyglucose (^18^F-FDG), the most commonly used PET tracer, shows non-specific periprosthetic increased uptake for as long as several years, even in patients without evidence of infection or loosening [20]. However, also ^18^F-Fluoride may show persisting non-specific uptake at the prosthesis/bone interface of total hip and total knee arthroplasties [Son et al.], which necessitated the definition of non-specific uptake patterns of ^18^F-Fluoride in our study to reduce the number of false-positive results.

The emphasis in our study was put on the differentiation between loosened and not loosened prostheses, the differentiation between septic and aseptic loosening was not in the focus of our study. A quantitative evaluation has not been performed so far and serves as an limitation of the current study. However dynamic quantitative evalutation might give an insight into differentiation between septic and aseptic loosening. To address this topic, the evaluation of dynamic ^18^F-Fluoride PET/CT scans with emphasis on an infection-related loosening will be part of further ongoing studies.

## PATIENTS AND METHODS

### Patient population

The study was approved of by the local institutional review board.

We included 26 patients in our retrospective study who suffered from painful total hip or total knee arthroplasties with radiological or clinical suspicion of peri-prosthetic loosening at least one year after implantation. Patients with prior oncologic disease have been excluded from the study. In the case of multiple inlaying prostheses without pain or clinical suspicion of loosening, the additional total knee and hip arthroplasties were also included into the study since the ^18^F-Fluoride PET/CT also produced images of these implants. All included patients were examined via ^18^F-Fluoride PET/CT between September 2014 and July 2017.

All patients were subject to routine clinical examination, laboratory and radiological studies. Data, such as development of pain and other clinical symptoms were gathered by chart review of medical records. No definite diagnosis had been established in any patient before the ^18^F-Fluoride PET/CT scan was performed. Revision operations were performed by experienced surgeons 1.4 ± 0.2 months after PET/CT scan. If no operation has been performed, the final evaluation and diagnosis was based on the long term clinical follow up of minimum 6 months (median: 11.3 months, range 6.0 – 29.7 months). In these cases the patient either refused to undergo revision surgery and showed further progression of the radiological signs of loosening or experienced spontaneous regression of symptoms. All patients were examined with informed consent. Both components of the total hip (acetabular and femoral part) and total knee arthroplasty (femoral and tibial part) were independently evaluated as true positive or true negative according to the intraoperative findings or long-term clinical follow up. 9 patients had two, and one patient had 3 inlaying prostheses.

### PET scanning

PET imaging was performed with a Biograph 2 PET/CT scanner (Siemens Healthcare GmbH, Erlangen, Germany) with an axial field of view of 16.2 cm and a transversal field of view of 58.5 cm. ^18^F-Fluoride was injected intravenously at a mean activity of 292 (± 24) MBq. Static emission scans of the legs were performed at a mean of 104 (± 18) min after injection (4 minutes emission time per bed position, 3D mode). PET images were reconstructed iteratively (4 iterations, 8 subsets, 256 × 256 matrix, 5mm slice thickness). A low-dose CT (16 mAs, 130 kV) was acquired for attenuation correction and anatomical correlation.

### Image interpretation

The scans were visually evaluated by an experienced nuclear medicine physician. The evaluation criteria were based on the pattern and location of the ^18^F-Fluoride uptake at the prosthesis/bone interface or cement/bone interface.

**Figure 1:**
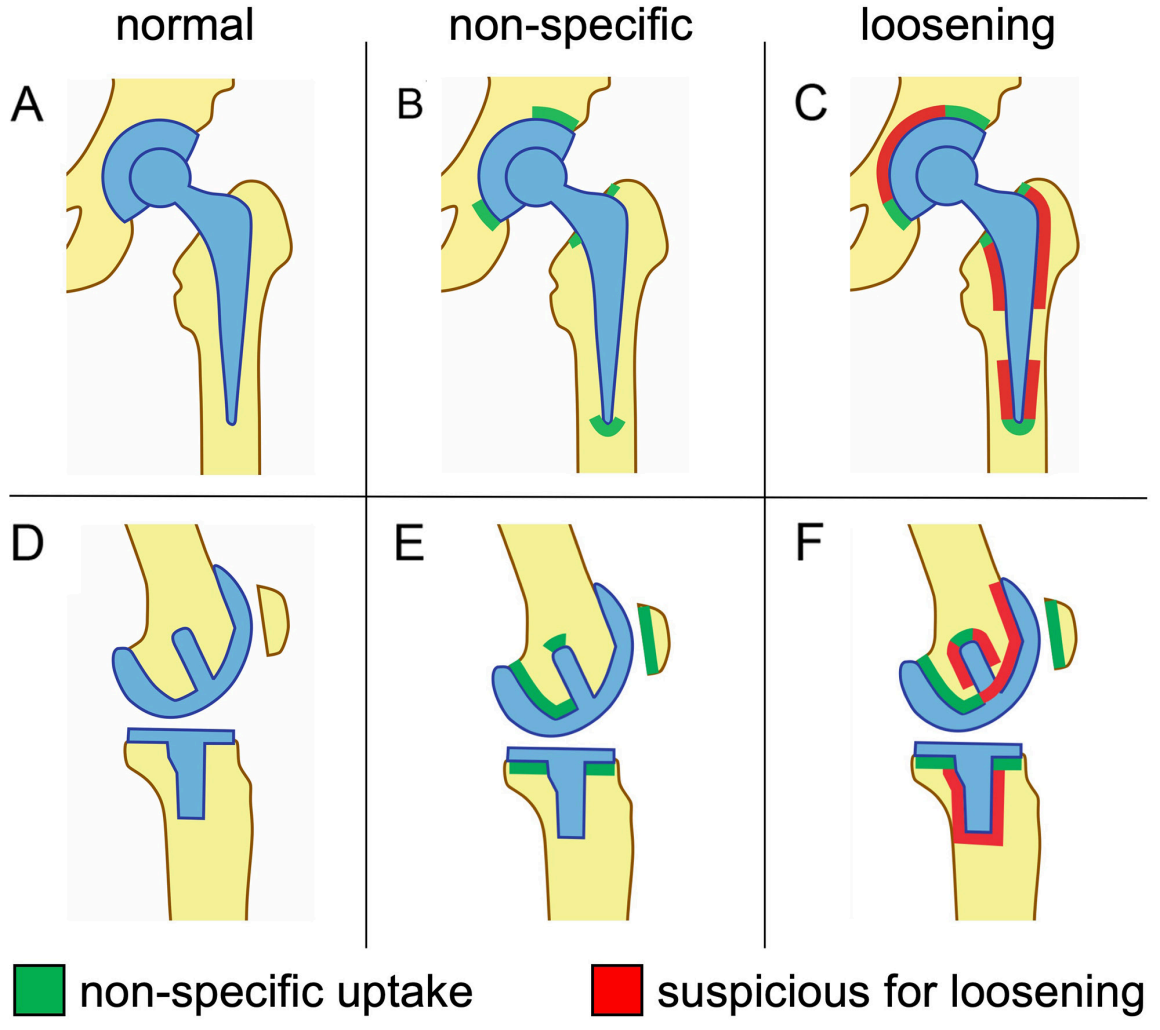
Schematic representation of ^18^F-Fluoride uptake patterns of total hip arthroplasties **(A-C)** and total knee arthroplasties **(D-F)** interpreted as non-specific **(B, E)** or suspicious for implant loosening **(C, F)**.

The criteria we used in our current study to differentiate non-specific uptake of ^18^F-Fluoride from patterns specific for loosening are in close correlation with uptake patterns described by Son et al., who previously examined postoperative ^18^F-Fluoride uptake in asymptomatic patients up to 25 months after hip or knee arthroplasty [21]. Our criteria for non-specific uptake correspond to areas of the bone interface which showed intense uptake in more than 75% of asymptomatic patients in the study by Son et al. (Normal or no uptake see Figure 1 (A,D)) Specifically, these are the cranial and caudomedial parts of the acetabular component and the distal tip of the femoral component of hip prostheses. Additionally, we categorized small areas of focal uptake in the proximal region of the femoral component as non-specific, as an increased frequency of persistent uptake of ^99m^Tc-HDP has been observed in these areas in a previous study in asymptomatic patients with uncemented hip prostheses by Kim et al. [22], see Figure 1B. Regarding total knee arthroplasty, in correlation with the data by Son et al., uptake along the dorsal and caudal parts of the femoral sleds was rated as non-specific, as well as uptake solely in the horizontal surfaces in the proximal region of the tibial component. These criteria also closely match the methods used in the study by Sterner et al. for the detection of aseptic loosening of total knee arthroplasties by ^18^F-Fluoride PET [14]. Additionally, we categorized uptake in the patellar region as non-specific, as well as small focal uptake at the tip of a pin/shaft of the femoral component, see Figure 1E.

Uptake exceeding these areas was interpreted as positive for loosening. Regarding hip prostheses, circular uptake at the interface encompassing more than half of the acetabular component was interpreted as positive for loosening, whereas for the femoral component, extended uptake around the trochanter regions or the distal parts of the shaft (exceeding the area of the tip) was rated positive (Figure 1C). With regard to total knee arthroplasties, uptake at the interface encompassing more than half of a condylar sled extending into the anterior parts, or extensive uptake around the pin/shaft of the femoral component was categorized as suspicious for loosening, as well as tracer accumulation around the pin/shaft of the tibial component (Figure 1F).

**Figure 2:**
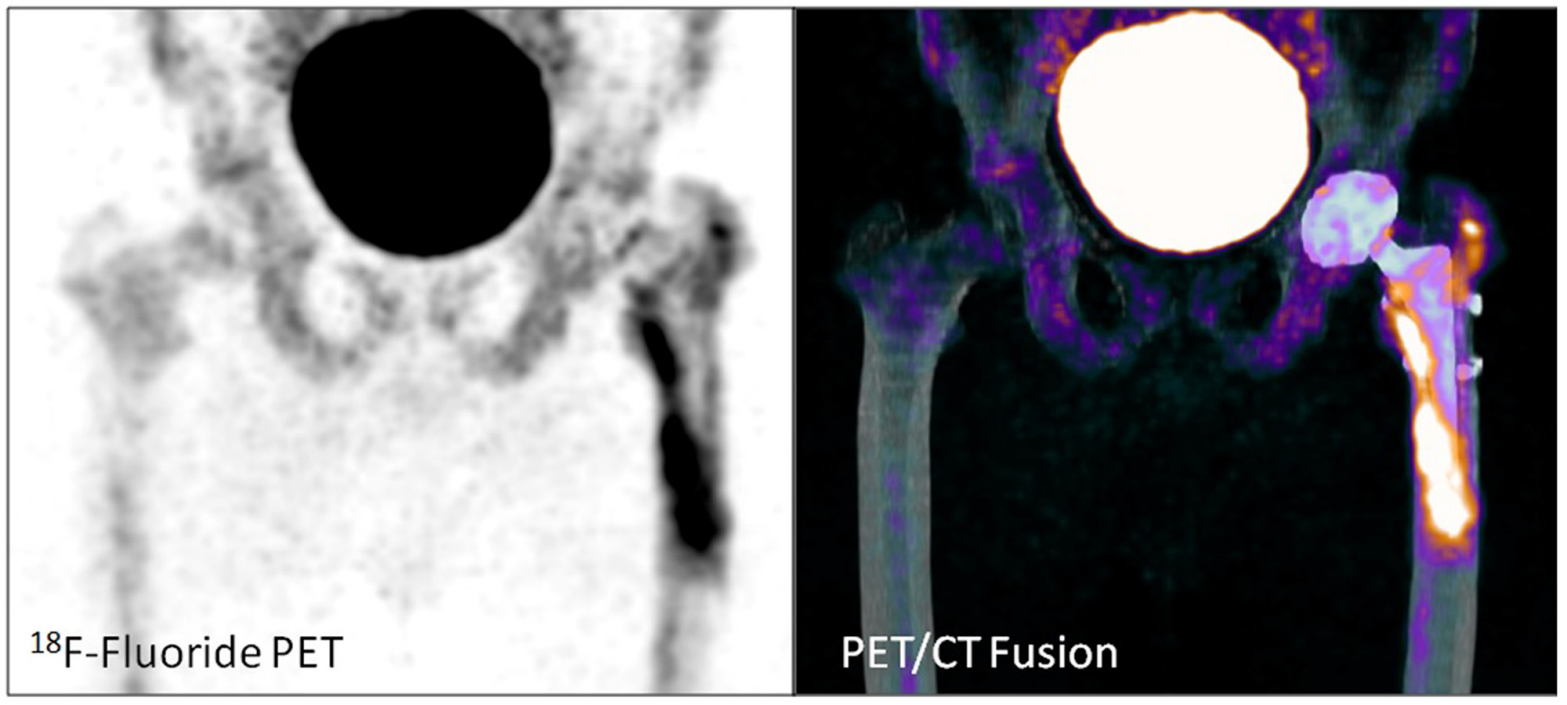
Total hip arthroplasty in a 55 year old male patient (patient number 14) 19 months after implantation. Loosening of the left femoral component (uptake along the stem including the tip). No signs of loosening of the left acetabular component.

**Figure 3:**
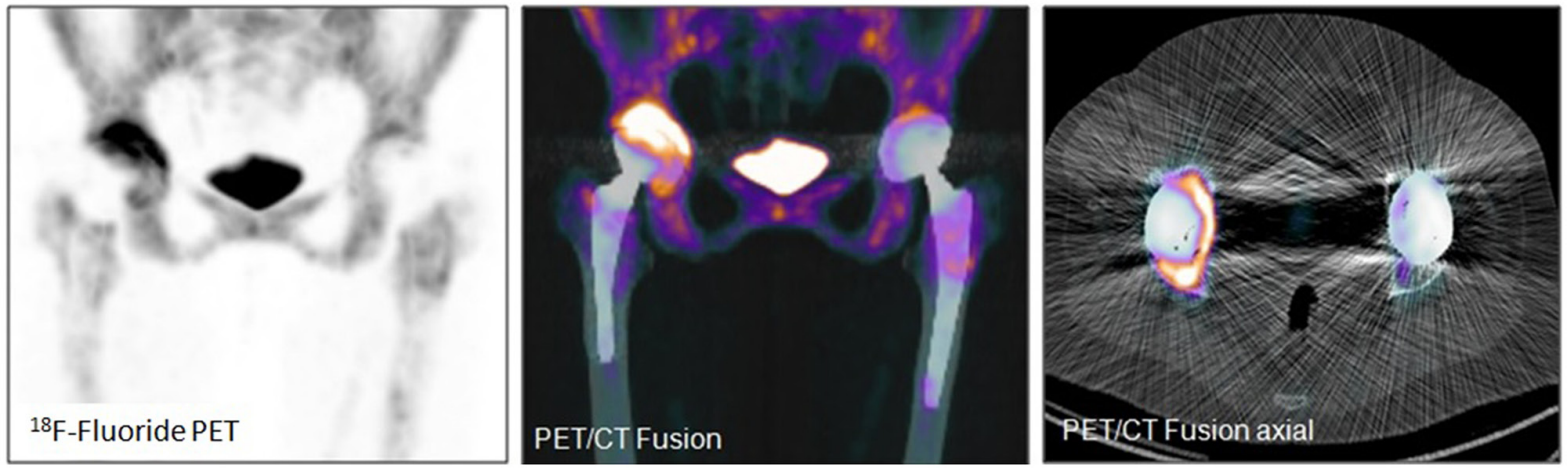
Total hip arthroplasty in a 72 year old female patient (patient number 2) 12.2 years after implantation. Loosening of the right acetabular component (circular uptake encompassing more than half of the acetabular component). No signs of loosening of the right femoral component, nor the left total hip arthroplasty.

**Figure 4:**
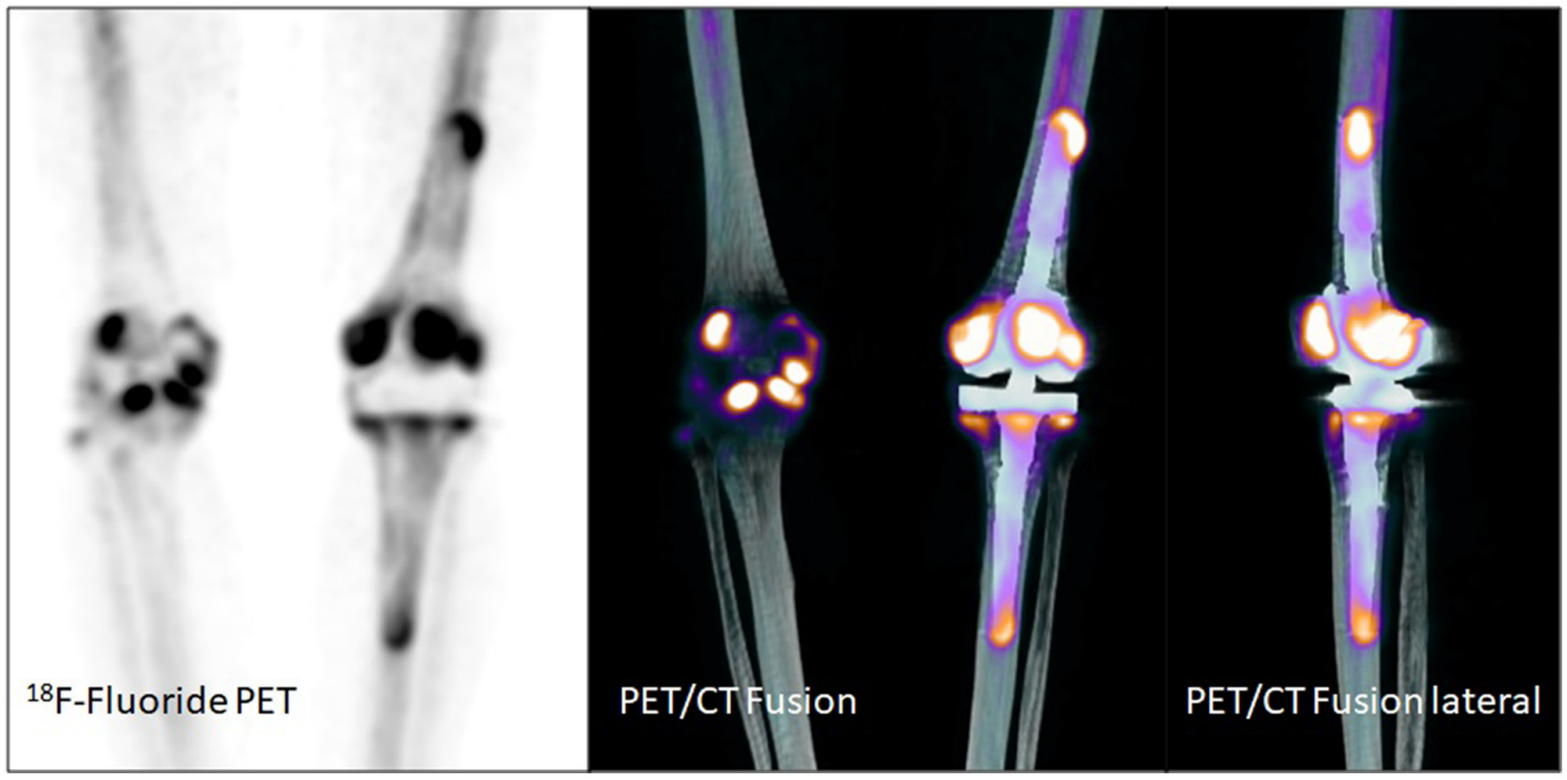
Total knee arthroplasty in a 67 year old female patient (patient number 15) 12 months after implantation. Suspicion of loosening of both components (femoral and tibial) of the total knee arthroplasty on the left side. Gonarthrosis on the right side.

**Figure 5:**
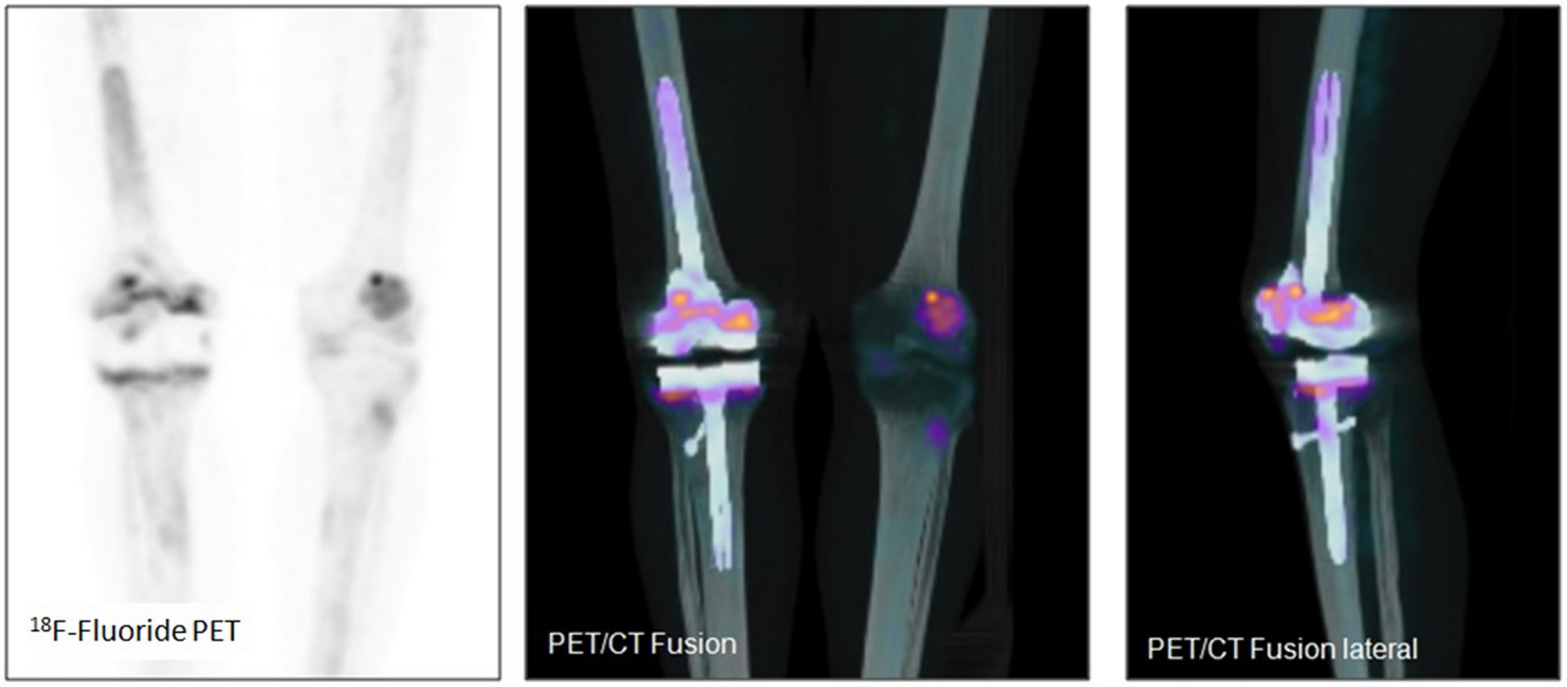
Total knee arthroplasty in an 82 year old male patient (patient number 22) 87 months after implantation. No signs of loosening, rather non-specific uptake along the weight bearing parts of the prosthesis.

Examples of patient examinations are shown in Figures 2, 3, 4, 5.

### Data analysis

The analogically recorded values out of the register were digitized and exported to MS Excel 2007 (Microsoft, Redmond, WA, USA) and SPSS 17 (IBM, Armonk, NY, USA) for further calculation. Subject of calculation by 2×2 table were sensitivity, specificity, accuracy.

## CONCLUSIONS

The results of our study show that ^18^F-Fluoride PET/CT is a useful and promising technique in diagnosing periprosthetic loosening of total hip and knee arthroplasties. It demonstrates a very high sensitivity and specificity, not only compared to those of other imaging tools such as traditional bone scan, but also to other published trials in this regard. Moreover, it shows potential to completely replace traditional bone scan in the diagnostic pathway of periprosthetic loosening. Due to the promising findings of this retrospective analysis, further prospective studies are warranted with the recruitment of a higher number of patients to define the role and usage of ^18^F-Fluoride PET/CT in routine clinical practice. Future studies should also focus on the uptake kinetics of ^18^F-Fluoride in periprosthetic joint infection to assess its value in distinguishing aseptic from septic loosening. Furthermore, the PET-scanning technique is also applicable with other tracers, such as ^68^Ga-zoledronate, which shows a slightly better availability than ^18^F-Fluoride and is therefore subject of a current prospective study of the same group.
